# Hyperuricemia treatment in acute heart failure patients does not improve their long‐term prognosis: A propensity score matched analysis from the AHEAD registry

**DOI:** 10.1002/clc.23197

**Published:** 2019-05-29

**Authors:** Marie Pavlusova, Jiri Jarkovsky, Klara Benesova, Jiri Vitovec, Ales Linhart, Petr Widimsky, Lenka Spinarova, Kamil Zeman, Jan Belohlavek, Filip Malek, Marian Felsoci, Jiri Kettner, Petr Ostadal, Cestmir Cihalik, Jiri Spac, Hikmet Al‐Hiti, Marian Fedorco, Richard Fojt, Andreas Kruger, Josef Malek, Tereza Mikusova, Zdenek Monhart, Stanislava Bohacova, Lidka Pohludkova, Filip Rohac, Jan Vaclavik, Dagmar Vondrakova, Klaudia Vyskocilova, Miroslav Bambuch, Gabriela Dostalova, Stepan Havranek, Ivana Svobodová, Ladislav Dusek, Jindrich Spinar, Roman Miklik, Jiri Parenica

**Affiliations:** ^1^ Department of Internal Medicine and Cardiology University Hospital Brno Brno Czech Republic; ^2^ Faculty of Medicine Masaryk University Brno Czech Republic; ^3^ Institute of Biostatistics and Analyses, Faculty of Medicine Masaryk University Brno Czech Republic; ^4^ First Department of Internal Medicine, Cardiology and Angiology St Anne's University Hospital Brno Brno Czech Republic; ^5^ Second Department of Internal Medicine, Department of Cardiology and Angiology First Faculty of Medicine of the Charles University, Prague, and General University Hospital in Prague Czech Republic; ^6^ University Hospital Kralovske Vinohrady and the Third Faculty of Medicine of the Charles University Prague Czech Republic; ^7^ Department of Internal Medicine Hospital Frydek‐Mistek Frydek‐Mistek Czech Republic; ^8^ Department of Cardiology Hospital Na Homolce Prague Czech Republic; ^9^ Department of Cardiology Institute of Clinical and Experimental Medicine Prague Czech Republic; ^10^ Department of Internal Medicine University Hospital Olomouc Olomouc Czech Republic; ^11^ Second Department of Internal Medicine St Anne's University Hospital Brno Brno Czech Republic; ^12^ Department of Internal Medicine Hospital Havlickuv Brod Havlickuv Brod Czech Republic; ^13^ Department of Internal Medicine Hospital Znojmo Znojmo Czech Republic; ^14^ Department of Cardiology Tomas Bata Regional Hospital Zlin Czech Republic; ^15^ Department of Internal Medicine Military Hospital Brno Brno Czech Republic

**Keywords:** acute heart failure, AHEAD, allopurinol

## Abstract

**Background:**

Hyperuricemia is associated with a poorer prognosis in heart failure (HF) patients. Benefits of hyperuricemia treatment with allopurinol have not yet been confirmed in clinical practice. The aim of our work was to assess the benefit of allopurinol treatment in a large cohort of HF patients.

**Methods:**

The prospective acute heart failure registry (AHEAD) was used to select 3160 hospitalized patients with a known level of uric acid (UA) who were discharged in a stable condition. Hyperuricemia was defined as UA ≥500 μmoL/L and/or allopurinol treatment at admission. The patients were classified into three groups: without hyperuricemia, with treated hyperuricemia, and with untreated hyperuricemia at discharge. Two‐ and five‐year all‐cause mortality were defined as endpoints. Patients without hyperuricemia, unlike those with hyperuricemia, had a higher left ventricular ejection fraction, a better renal function, and higher hemoglobin levels, had less frequently diabetes mellitus and atrial fibrillation, and showed better tolerance to treatment with angiotensin‐converting enzyme inhibitors/angiotensin receptor blockers and/or beta‐blockers.

**Results:**

In a primary analysis, the patients without hyperuricemia had the highest survival rate. After using the propensity score to set up comparable groups, the patients without hyperuricemia had a similar 5‐year survival rate as those with untreated hyperuricemia (42.0% vs 39.7%, *P* = 0.362) whereas those with treated hyperuricemia had a poorer prognosis (32.4% survival rate, *P* = 0.006 vs non‐hyperuricemia group and *P* = 0.073 vs untreated group).

**Conclusion:**

Hyperuricemia was associated with an unfavorable cardiovascular risk profile in HF patients. Treatment with low doses of allopurinol did not improve the prognosis of HF patients.

**ABBREVIATIONS**
ACEI: angiotensin converting enzyme inhibitor
AHEAD: Acute HEArt failure Database
ARB: angiotensin receptor blocker
eGFR: estimated glomerular filtration rate
HF: heart failure
NO: nitric oxide
ROA: reactive oxygen species
UA: uric acid
XO: xanthine oxidase

## 1 INTRODUCTION

Heart failure (HF) affects over 23 million people worldwide. Despite improvements in both pharmacological and non‐pharmacological treatment, its prognosis remains rather poor: patients hospitalized for acute heart failure have 1‐ and 5‐year mortality rates 32% and 60%, respectively.^1^ The current European Society of Cardiology (ESC) guidelines for HF highlight the importance of comorbidities that might influence the patients' prognosis and quality of life.^2^ Hyperuricemia might be an extremely interesting culprit in HF and cardiovascular disease. Several publications have recently identified hyperuricemia as an independent unfavorable prognostic factor in HF patients.^3^, ^4^ Elevated levels of uric acid (UA) have been reported in up to 50% of HF patients, which is in stark contrast to 2% to 18% of people in a healthy population.^5^ The pathophysiological association between elevated levels of UA and an unfavorable prognosis of HF patients has not been clarified yet. UA is the end product of purine metabolism. In a two‐step reaction, the enzyme xanthine oxidase (XO) catalyzes the oxidation of hypoxanthine to xanthine and the oxidation of xanthine to UA. During this process, a highly reactive superoxide radical (O_2_^−^) ‐ one of the reactive oxygen species (ROS) ‐ is produced. There are several possible contributors to elevated levels of UA in HF, including increased activity of XO or decreased renal excretion of UA.^5^ Diuretic therapy can also contribute to elevated levels of UA.^6^

The elevation of UA levels itself can have a negative prognostic influence, but the up‐regulation of XO activity is probably a significant contributor, too. Elevated UA levels are associated with a reduction in nitric oxide (NO) levels and with endothelial dysfunction.^7^ On the other hand, UA has an antioxidant effect and might counteract the effect of ROS.^8^, ^9^

To some extent, hyperuricemia is a marker of upregulation of XO activity, which subsequently leads to an increase in oxidative stress. Oxidative stress plays an important negative role in the pathophysiology of HF: it causes myocardial fibrosis,^10^ left ventricular remodeling,^11^ decreased myocardial contractility,^12^ and diastolic dysfunction.^13^ In a mouse model, XO inhibition by allopurinol delayed heart failure progression.^14^ In HF patients, inhibition of XO by allopurinol had a beneficial effect in terms of improved endothelial dysfunction,^15^ decreased levels of B‐type natriuretic peptide^16^ and improved left ventricular contractility.^17^, ^18^ On the other hand, two smaller randomized trials in patients with left ventricular systolic dysfunction did not prove a positive clinical effect of hyperuricemia treatment with allopurinol.^8^, ^19^

Hyperuricemia is therefore a marker of poor prognosis in HF patients. There are experimental data on the beneficial effect of XO inhibition by allopurinol but there is no evidence on the benefit of this treatment in clinical practice.

Using the propensity score, we examined data from the AHEAD registry to assess whether treatment of hyperuricemia with allopurinol is associated with improvements in middle and long‐term mortality in acute HF patients.

## 2 METHODS

### 2.1 Study population

**Figure 1:**
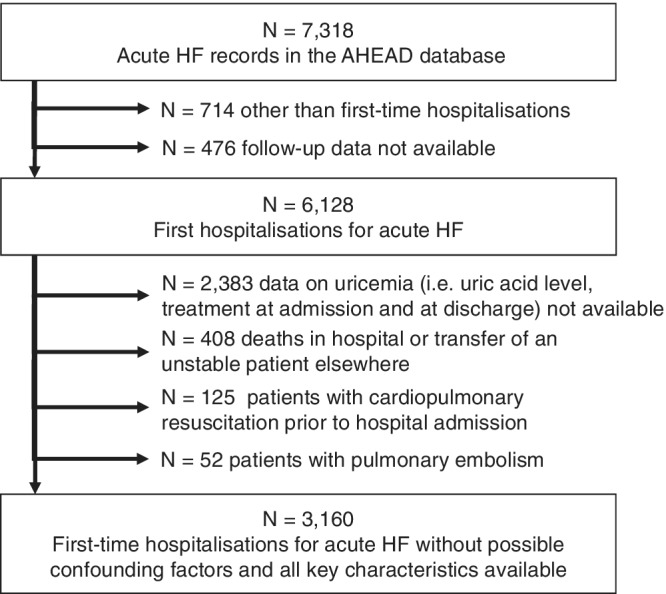
Flow chart of data analysis

The AHEAD registry, with inclusion and exclusion criteria, was described in detail in previous publications.^20^, ^21^ In brief, patients hospitalized with acute HF in 15 centers across a region with approximately 3 million inhabitants in the Czech Republic were consecutively enrolled between 2006 and 2012 and followed after their discharge. A total of 7318 records on hospitalizations for acute HF were prospectively collected in the registry. In our analyses, we only included records on HF patients who were hospitalized for the first time for HF, regardless of whether they had de novo heart failure or acute decompensation of pre‐existing chronic HF, and on which follow‐up data were available. We excluded patients with unknown UA levels, those who died during their hospital stay or were transferred elsewhere in an unstable condition, those who were resuscitated before hospital admission and those with pulmonary embolism (Figure 1).

Mortality data were obtained from the centralized database of the Ministry of Health of the Czech Republic. At the time of evaluation, all patients were followed up for at least 5 years. Based on a previous study, prognostically unfavorable hyperuricemia was defined as UA ≥500 μmoL/L (8.41 mg/dL).^22^ The patients were classified into three groups: those without hyperuricemia (UA < 500 μmoL/L and not treated with allopurinol at admission), those with hyperuricemia and treated with allopurinol (UA > 500 μmoL/L and/or treated with allopurinol at admission; treated with allopurinol at discharge) and those with untreated hyperuricemia (UA > 500 μmoL/L and/or treated with allopurinol at admission; not treated with allopurinol at discharge).

The 24‐month all‐cause mortality was the primary endpoint and the 5‐year all‐cause mortality was the secondary endpoint. This study was carried out in accordance with the 1975 Declaration of Helsinki and was approved by the Ethics Committee of the University Hospital Brno (Brno, Czech Republic). All patients were enrolled in the study upon providing written informed consent.

### 2.2 Statistical methods

Baseline patient characteristics were described using absolute and relative frequencies for categorical variables and median values supplemented with 5th‐95th percentile range for continuous variables. Factors previously demonstrated as significant for the prognosis were evaluated (Table 1).

**Table 1:** Patient characteristics before propensity score matching

	Total	No hyperuricemia	Hyperuricemia, treated	Hyperuricemia, untreated	*P*‐value
No. of patients	3160	1785	793	582	
Age	73 (64; 80)	73 (64; 80)	73 (65; 80)	74 (64; 80)	0.657
Sex: woman	1295 (41.0%)	783 (43.9%)	286 (36.1%)	226 (38.8%)	**<.001**
BMI	28 (25; 32)	28 (25; 31)	29 (26; 34)	29 (25; 32)	**<.001**
SBP	140 (120; 160)	140 (120; 160)	140 (120; 160)	140 (110; 160)	**<.001**
EF (%)	38 (27; 50)	40 (30; 50)	35 (25; 50)	35 (25; 49)	**<.001**
eGFR (CKDEPI)	53 (38; 70)	60 (46; 77)	44 (30; 60)	43 (31; 57)	**<001**
Hemoglobin (g/L)	133 (118; 145)	134 (121; 146)	130 (114; 143)	132 (115; 145)	**<.001**
Uric acid (μmol/L)	413 (330; 506)	370 (302; 428)	503 (385; 604)	563 (520; 626)	**<.001**
NT‐proBNP (pg/mL)	4558 (2337; 9353)	3763 (1911; 8076)	5263 (3075; 9101)	6414 (2974; 12 728)	***<*.001**
Killip class – III + IV	829 (26.2%)	473 (26.5%)	164 (20.7%)	192 (33.0%)	**<.001**
Atrial fibrillation	961 (30.4%)	477 (26.7%)	296 (37.3%)	188 (32.3%)	**<.001**
Diabetes mellitus	1449 (45.9%)	729 (40.8%)	420 (53.0%)	300 (51.5%)	**<.001**
History of CAD	1880 (59.5%)	1140 (63.9%)	409 (51.6%)	331 (56.9%)	**<.001**
ACEIs/ARBs	2612 (82.7%)	1502 (84.1%)	664 (83.7%)	446 (76.6%)	**<.001**
Beta‐blockers	2561 (81.0%)	1467 (82.2%)	653 (82.3%)	441 (75.8%)	**.002**
Diuretics	2699 (85.4%)	1433 (80.3%)	752 (94.8%)	514 (88.3%)	**<.001**

*Note*: Continuous variables are described by median values (IQR); categorical variables are described by absolute and relative frequencies. *P*‐value of Kruskal‐Wallis test for continuous variables and *P‐*value of the Fisher's exact test for categorical variables are reported for the comparison of patient characteristics according to the presence of hyperuricemia and its treatment. NT‐proBNP levels were only available in about 30% of patients.

Abbreviations: ACEIs, angiotensin‐converting enzyme inhibitors; ARBs, angiotensin receptor blockers; BMI, body mass index; CAD, coronary artery disease; EF, ejection fraction; eGFR, estimated glomerular filtration rate; SBP, systolic blood pressure.

*P* values of less than 0.05 (**in bold**) are statistically significant.

Statistical significance of differences in patient characteristics according to UA levels and hyperuricemia treatment (no hyperuricemia, hyperuricemia treated, hyperuricemia untreated) were tested using Kruskal‐Wallis test for continuous variables and Fisher's exact test for categorical variables. To achieve fair comparability among the patient groups, we used the propensity score based on logistic regression model including age, gender, body mass index, systolic blood pressure, left ventricular ejection fraction, estimated glomerular filtration rate (eGFR), hemoglobin, Killip class, atrial fibrillation, diabetes mellitus, history of coronary artery disease, and angiotensin‐converting enzyme inhibitors / angiotensin II receptor blockers (ACEIs/ARBs), beta‐blockers and diuretics at discharge. The uricemia‐untreated group was adopted as reference. The Kaplan‐Meier methodology was used for the assessment and visualization of a long‐term survival. Differences in survival between patient groups were tested by the log‐rank test.

The level of significance was set at α = 0.05 for all analyses. IBM SPSS 25.0.0.1 (IBM Corporation 2018) and R 3.5.1 with MatchIt package were used for the analysis.

## 3 RESULTS

### 3.1 Baseline characteristics

The mean age of patients was 73 years (64;80) and 41.0% of them were women. The mean ejection fraction was 38%; the proportions of patients with diabetes mellitus and a history of coronary artery disease were 45.9% and 59.5%, respectively. At discharge, 82.7%, 81%, and 85.4% of patients were treated with ACEIs/ARBs , beta‐blockers, and diuretics, respectively.

Hyperuricemia was observed in 43.5% of patients. Median levels of UA were 370 μmoL/L, 503 μmoL/L, and 563 μmoL/L in groups of patients without hyperuricemia, those with treated hyperuricemia and those with untreated hyperuricemia, respectively. Patients without hyperuricemia, unlike those with treated or untreated hyperuricemia, were more frequently women (43.9%, 36.1%, and 38.8%, respectively), had a higher left ventricular ejection fraction (40%, 35%, and 35%), better renal function (eGFR: 60, 44 and 43 mL/min/1.73 m^2^) and higher hemoglobin levels (134, 130 and 132 g/L), had less frequently diabetes mellitus (40.8%, 53.0%, and 51.5%) and atrial fibrillation (26.7%, 37.3%, and 32.3%). At discharge, patients without hyperuricemia were treated with diuretics less frequently than those with treated or untreated hyperuricemia (80.3%, 94.8%, and 88.3%). NT‐proBNP levels were known in approximately 30% of patients. Median levels of NT‐proBNP were 3763 pg/mL, 5263 pg/mL, and 6414 pg/mL (*P* < 0.001) in patients without hyperuricemia, with treated and untreated hyperuricemia, respectively.

**Figure 2:**
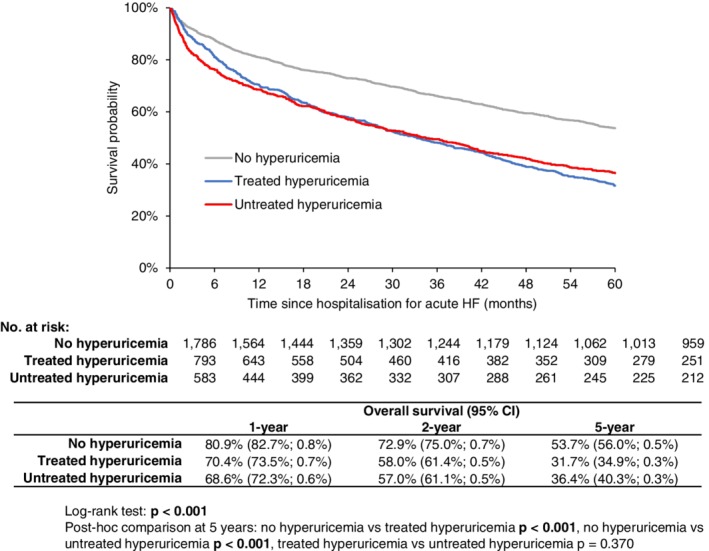
Kaplan‐Meier estimate of 5‐year overall survival in patients with acute heart failure according to hyperuricemia and its treatment (before propensity score matching)

In the unbalanced dataset, 2‐ and 5‐year survival rates among patients without hyperuricemia were significantly higher than in patients with hyperuricemia, regardless of whether the condition was treated with allopurinol or not: patients without hyperuricemia, with treated hyperuricemia and untreated hyperuricemia had 2‐year survival rates of 72.9%, 58.0%, and 57.0% (*P* < 0.001), respectively, and 5‐year survival rates of 53.7%, 31.7%, and 36.4%, respectively (Figure 2).

**Figure 3:**
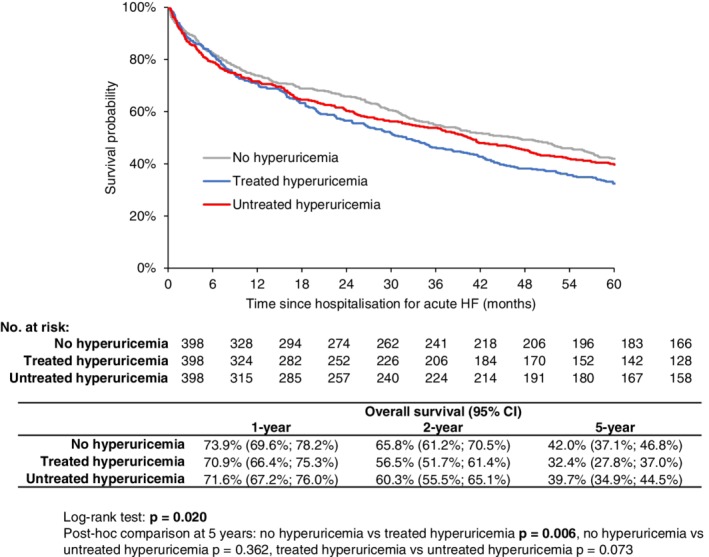
Kaplan‐Meier estimate of 5‐year overall survival in patients with acute heart failure according to hyperuricemia and its treatment (after propensity score matching)

Using a propensity score based on 14 prognostically important parameters identified in a previous paper,^21^ three balanced groups with comparable characteristics were set up (Table 2). NT‐proBNP levels were not included as a parameter in the propensity score because it was only known in a small subset of patients; despite that, it is evident that even this parameter, which reflects the severity of heart failure, was comparable in the balanced groups. Kaplan‐Meier curves for the balanced groups (Figure 3) revealed that the 1‐year all‐cause mortality was entirely comparable, but in the long‐term follow‐up the patients without hyperuricemia had a lower all‐cause mortality (*P* = 0.006) than the patients with treated hyperuricemia and there was a strong trend towards a better survival of the untreated hyperuricemia group when compared to the treated hyperuricemia group (*P* = 0.073).

**Table 2:** Patient characteristics after propensity score matching

	Total	No hyperuricemia	Hyperuricemia, treated	Hyperuricemia, untreated	*P*‐value
No. of patients	1194	398	398	398	
Age	73 (65; 80)	73 (65; 79)	73 (65; 81)	73 (65; 80)	.641
Sex: woman	441 (36.9%)	147 (36.9%)	147 (36.9%)	147 (36.9%)	1.000
BMI	29 (26; 33)	29 (26; 33)	29 (26; 33)	29 (26; 32)	.742
SBP	140 (115; 160)	135 (115; 160)	140 (120; 160)	140 (111; 160)	.609
EF (%)	35 (25; 47)	35 (25; 45)	35 (25; 50)	35 (25; 50)	.553
eGFR (CKDEPI)	46 (34; 61)	48 (36; 61)	46 (32; 62)	43 (33; 59)	.075
Hemoglobin (g/L)	132 (116; 145)	132 (116; 145)	131 (116; 145)	132 (117; 144)	.990
Uric acid (μmol/L)	468 (372; 565)	389 (322; 444)	484 (373; 601)	560 (519; 625)	**<.001**
NT‐proBNP (pg/mL)	5537 (2740; 10 915)	4867 (2402; 12 036)	4993 (3083; 8776)	6323 (2883; 11 918)	.642
Killip class – III + IV	328 (27.5%)	115 (28.9%)	100 (25.1%)	113 (28.4%)	.435
Atrial fibrillation	390 (32.7%)	125 (31.4%)	140 (35.2%)	125 (31.4%)	.435
Diabetes mellitus	618 (51.8%)	199 (50.0%)	212 (53.3%)	207 (52.0%)	.658
History of CAD	679 (56.9%)	225 (56.5%)	224 (56.3%)	230 (57.8%)	.904
ACEIs/ARBs	986 (82.6%)	330 (82.9%)	334 (83.9%)	322 (80.9%)	.523
Beta‐blockers	993 (83.2%)	337 (84.7%)	331 (83.2%)	325 (81.7%)	.550
Diuretics	1106 (92.6%)	366 (92.0%)	371 (93.2%)	369 (92.7%)	.809

Note*:* Continuous variables are described by median values (IQR); categorical variables are described by absolute and relative frequencies. *P*‐value of Kruskal‐Wallis test for continuous variables and *P*‐value of the Fisher's exact test for categorical variables are reported for the comparison of patient characteristics according to the presence of *hyperuricemia* and its treatment. NT‐proBNP levels were only available in about 30% of patients.

Abbreviations: ACEIs, angiotensin‐converting enzyme inhibitors, ARBs, angiotensin receptor blockers, BMI, body mass index, CAD, coronary artery disease, EF, ejection fraction, eGFR, estimated glomerular filtration rate, SBP, systolic blood pressure.

*P* values of less than 0.05 (**in bold**) are statistically significant.

## 4 DISCUSSION

The present study highlights three extremely important points regarding the care for heart failure patients: (a) hyperuricemia was a predictor of a poorer long‐term prognosis, (b) treatment with allopurinol, that is, xanthine oxidase inhibitor, had no influence on the patients' prognosis, (c) after setting up comparable groups using the propensity score, no difference in long‐term all‐cause mortality was observed between patients without hyperuricemia and those with untreated hyperuricemia. Two years and later after discharge, the mortality was significantly worse in patients with treated hyperuricemia than in those without hyperuricemia; the difference was not significant when compared treated hyperuricemia patients to patients with untreated hyperuricemia.

The admission characteristics of patients clearly show that patients without hyperuricemia had a more favorable profile of prognostic factors: higher left ventricular ejection fraction, better renal function, higher hemoglobin levels, lower occurrence of diabetes mellitus, and atrial fibrillation, better tolerance of treatment with ACEIs/ARBs and/or beta‐blockers. All of these parameters were associated with a better long‐term prognosis. Additionally, NT‐proBNP levels, which were only available in 30% of evaluated patients, were also lowest in patients without hyperuricemia, and highest in patients with untreated hyperuricemia. Our results suggest that elevated UA levels themselves are an indicator of a more serious condition in terms of prognosis rather than a risk factor which should be treated with the intention to improve the long‐term prognosis.

Our results are in agreement with several previous publications. The randomized double‐blind trial EXACT‐HF (Xanthine Oxidase Inhibition for Hyperuricaemic Heart Failure Patients), which followed up a group of 128 HF with reduced ejection fraction patients treated with 600 mg of allopurinol for 24 weeks, did not prove any effect on mortality and/or deterioration of HF when compared to the control group. Allopurinol treatment did not lead to a change in NT‐proBNP levels either, although uric acid levels dropped by almost 50%.^23^ Likewise, clinical effect of this treatment was not proved in a retrospective analysis of chronic HF patients who were treated with 100 and 300 mg doses of allopurinol^24^ or in patients with gout.^25^ On the other hand, some studies have suggested that allopurinol treatment might have a beneficial effect on the all‐cause mortality in the general population^26^ and on the cardiovascular mortality in patients with gout.^27^, ^28^ Only a higher dose of allopurinol (≥300 mg) might have a protective effect against the progression of renal dysfunction in patients with hyperuricemia.^29^, ^30^

The results of our study bring important information for further research as well as for care for patients with HF and hyperuricemia: generally administered xanthine oxidase inhibition by allopurinol at a low dose of 100 mg has no impact on the prognosis of HF patients with hyperuricemia (UA ≥500 μmoL/L). Despite findings of the above‐mentioned studies, there is still not enough evidence to substantiate treatment of asymptomatic hyperuricemia with higher doses of allopurinol (300 mg/day). According to the current HF guidelines (and in agreement with the European League Against Rheumatism guidelines),^2^, ^31^ hyperuricemia treatment with XO inhibitors (allopurinol, oxypurinol) is indicated in patients with gout, with the aim of achieving UA levels <357 μmoL/L.

Our study has several limitations. Only all‐cause mortality was available, not cardiovascular mortality in particular. Our analysis is based on a one‐time determination of UA levels and on hyperuricemia treatment at the time of discharge from hospital. After discharge, changes in allopurinol therapy might have occurred. Decrease in UA levels was not monitored, so we were not able to evaluate the effect of allopurinol treatment. Patients were mostly treated with allopurinol at a low dose of 100 mg. Only allopurinol‐based treatment was evaluated; a more recent approach to hyperuricemia treatment ‐ such as with probenecid, which might potentially decrease the risk of cardiovascular events^32^ ‐ was not evaluated. A large cohort of consecutive patients, in which patients with HF with reduced ejection fraction, those with EF 40% to 49% and those with EF ≥50% (heart failure with preserved ejection fraction) were involved, was the advantage of our study. Moreover, the patients were followed up for a minimum of 5 years.

## 5 CONCLUSION

Hyperuricemia was associated with an unfavorable cardiovascular risk profile in HF patients. Treatment of hyperuricemia with low doses of allopurinol did not improve the long‐term prognosis of HF patients.

## CONFLICT OF INTEREST

The authors declare no potential conflict of interests.

## AUTHOR CONTRIBUTIONS

All authors take responsibility for all aspects of the reliability and freedom from bias of the data presented and their discussed interpretation and approved their publication. All authors contributed to the conception and design of the study, data collection and interpretation of the results, and to writing or revising the manuscript. Marie Pavlusova, Roman Miklik, Jiri Jarkovsky, and Jiri Parenica are contributed to the conceptualization and validation. Jiri Jarkovsky, Marian Felsoci, Jindrich Spinar, and Jiri Parenica are contributed to the methodology. Jiri Jarkovsky, Klara Benesova, Ladislav Dusek are contributed to formal analysis. Marie Pavlusova, Kamil Zeman, Marian Felsoci, Marian Fedorco, Richard Fojt, Andreas Kruger, Josef Malek, Tereza Mikusova, Stanislava Bohacova, Lidka Pohludkova, Filip Rohac, Dagmar Vondrakova, Klaudia Vyskocilova, Miroslav Bambuch, Gabriela Dostalova, Stepan Havranek, Ivana Svobodová, and Roman Miklik are contributed to the investigation. Jiri Parenica and Jindrich Spinar are contributed to the resources and funding. Jiri Jarkovsky, Klara Benesova, Roman Miklik, and Jiri Parenica are contributed to the data cleaning. Marie Pavlusova, Roman Miklik, and Jiri Parenica are contributed to the writing and original draft preparation. Jan Vaclavik, Ales Linhart, Petr Widimsky, Lenka Spinarova, Jan Belohlavek, Filip Malek, Jiri Kettner, Petr Ostadal, Cestmir Cihalik, Jiri Spac, Hikmet Al‐Hiti, Zdenek Monhart, Jan Vaclavik, and Jindrich Spinar are contributed to the review and editing the manuscript. Jiri Jarkovsky, Klara Benesova, Ladislav Dusek, Roman Miklik are contributed to the visualization. Roman Miklik and Jiri Parenica are contributed to the supervision.
